# iTRAQ-mediated analysis of the relationship between proteomic changes and yak *longissimus lumborum* tenderness over the course of postmortem storage

**DOI:** 10.1038/s41598-021-90012-0

**Published:** 2021-05-17

**Authors:** Yayuan Yang, Jieyuan Yang, Jibing Ma, Qunli Yu, Ling Han

**Affiliations:** 1 Key Laboratory of Veterinary Pharmaceutical Development, Ministry of Agricultural and Rural Affairs, Lanzhou Institute of Husbandry, Pharmaceutical Sciences of Chinese Academy of Agricultural Sciences, Lanzhou, 730050 People’s Republic of China; 2 College of Food Science and Engineering, Gansu Agricultural University, 1#, Yingmen Village, Anning, Lanzhou, 730070 Gansu People’s Republic of China; 3 School of New Energy and Power Engineering, Lanzhou Jiaotong University, Lanzhou, 730070 Gansu People’s Republic of China

**Keywords:** Enzyme mechanisms, Proteins, Animal physiology

## Abstract

To identify differentially expressed proteins associated with energy metabolism and tenderness during the postmortem aging of yak longissimus lumborum muscle samples, we collected tissue samples from yaks raised at different altitudes. At 12 h post-slaughter, we identified 290 differentially expressed proteins (DEPs) in these samples, whereas 436 such DEPs were detected after 72 h. Identified DEPs were clustered into four main functional categories: cell structural proteins, glycogen metabolic proteins, energy reserve metabolic proteins, and cellular polysaccharide metabolic proteins. Further bioinformatics analysis revealed that these proteins were associated with carbon metabolism, glycolysis, and the biosynthesis of amino acids. Our functional insights regarding these identified proteins contribute to a more detailed molecular understanding of the processes of energy metabolism in yak muscle tissue, and represent a valuable resource for future investigations.

## Introduction

To date, the majority of scientific analyses aimed at evaluating meat quality have primarily focused on tenderness and its impact on consumer perceptions of quality. In addition to being closely linked to meat quality, meat tenderness is also influenced by the levels and activities of many different proteins within the muscles, wherein they act to regulate a variety of biological activities including proteolysis and structural alterations. Importantly, the expression and activity of these proteins can influence meat tenderness and quality over time during post-mortem storage prior to consumer consumption.

Hypoxic conditions can cause mammalian cells and tissues to express a number of different glucose transporters and enzymes that ultimately lead them to shift their metabolic activity such that they favor anaerobic glycolysis rather than aerobic respiration, leading to ATP generation that coincides with the reduced production of toxic reactive oxygen species (ROS)^1^. In rat heart tissue, for example, acute hypoxia can result in a significant increase in phosphofructokinase (PFK) activity and lactate production together with a net reduction in cardiac ATP levels^2^. Comparable findings have also been made in different studies of yak breeds that reside between 1500 and 5000 m of elevation in southwestern China^3–5^. As they are adapted to high altitude conditions, yaks exhibit more robust energy metabolism and superior disease resistance relative to other cattle species. Importantly, under low oxygen conditions yaks of the Tibetan Plateau are able to readily induce the expression of certain proteins that allow their cells to better adapt to hypoxia so as to improve circulation and preserve cellular functionality. We have previously shown that yaks raised at high altitudes exhibit significantly reduced tissue glycogen levels that coincide with significant increases in hemoglobin and lactate levels in blood samples from these same animals^6^. Cooking loss of the longissimus dorsi of low altitude Simmental cattle is lower than that of Yushu yak and Gannan yak, but the difference is not significant. The L* and b* values of Simmental are slightly lower than Yushu yak and Gannan yak, and the a* value is higher than Yushu yak and Gannan yak. Cooking loss of the three groups first increased significantly with the maturation time (0–72 h) (*P* < 0.05), and the Yushu Yak, Gannan yak, and Simmental cattle reached the maximum at 72 h after slaughter, respectively^7^. In order to adapt to the cold and high altitude environment. Yushu yak must have enough energy supply to maintain body temperature. Anaerobic metabolism (glycolysis) is inefficient. Each glucose molecule produces only two ATP molecules, while complete oxidative metabolism produces 38 ATP molecules. Therefore, Yushu yak must improve energy production efficiency in order to reduce high-altitude breeds to increase oxidative metabolism and reduce anaerobic metabolism. According to the above research, the glycolysis potential of Yushu yak is lower than that of Gannan yak, which may lead to their superior meat quality characteristics than Simmental cattle^8^. These findings clearly show that hypoxic conditions can alter the metabolic state within a given animal. How different altitudes impact hypoxia resistance in yaks, however, has not been thoroughly studied. In this analysis, we therefore examined the relationship between hypoxia resistance and energy metabolism in yaks from different altitudes, and we further explored the mechanisms underlying this relationship.

The iTRAQ proteomics system is a promising and quantitative approach that allows for the identification of biomarkers of particular physiological or pathological states, as it facilitates high proteomic coverage with a large dynamic range^9^. Despite this promise, however, no previous studies have conducted an iTRAQ-based proteomic assessment of factors associated with *longissimus lumborum* tenderness. In the present study, we therefore utilized the iTRAQ technology to identify differentially expressed biomarkers of tenderness in yak longissimus lumborum samples over a defined period of postmortem storage. Using protein bioinformatics strategies, we then further explored the mechanistic basis for the tenderization of these *longissimus lumborum* samples.

In this analysis, we specifically focused on comparing the iTRAQ proteomic profiles of longissimus lumborum samples from Yushu and Gannan yaks that had been raised at different altitudes. As such, our findings offer insight into the proteomic basis for high-altitude adaptations in these animals. Such profiling efforts have the potential to shed new light on hypoxic adaptations and the proteins associated therewith. In addition to this proteomic profiling effort, we additionally conducted gene ontology (GO) and Kyoto encyclopedia of genes and genomes (KEGG) pathway enrichment analyses, and explored putative protein–protein interactions among differentially expressed proteins in an effort to identify central hub proteins associated with our phenotypes of interest. Through this approach, we highlighted novel molecular mechanisms linked to yak muscle tenderness.

## Materials and methods

### Materials

All *M. longissimus lumborum* (LL, the anterior 12th rib to the last lumbar vertebrae) samples were collected from 10 yaks from Yushu Tibetan Autonomous Prefecture (Altitude: 4500 m, Longitude: 97.008762, Latitude 33.00393), Qinghai Province, China and 10 yaks from the Tibetan Autonomous Prefecture of Gannan (Altitude: 2500 m, Longitude: 102.0754, Latitude: 33.997), with animals having a live weight of about 240–280 kg. All animals were of similar age, and the two groups had similar feeding and carcass conditions. The experiment was carried out in October. The protocol and procedures adopted in the next operation received the review and approval from Institutional Animal Care and Use Committee of Gansu Agricultural University (Approved ID: 2012-2-159). Briefly, the cattle should have a full rest for 12–24 h and stop water supply form the first 3 h before being slaughtered. After the cattle were sent to the slaughter site, their esophagus, tracheas and blood vessels were synchronously cut off from the larynxes and drained of blood. The carcasses of the cattle were then hung upside down and their cowhides, hooves, heads and internal organs were removed in turn. These samples were removed immediately after slaughtering, vacuum packed, and transported to our laboratory at 3 ± 1 °C. Additional samples were snap-frozen for 5 min in liquid nitrogen as 0 h samples. Fat was carefully removed from the *M. longissimus lumborum*, and each sample was then sliced into 150 ± 10 g sub-samples that were transferred to a pallet and stored at 3 ± 1 °C for up to 72 h. Samples of LL tissue were taken during the postmortem period at three different time points (0, 12, and 72 h). Samples were washed with PBS to remove any blood and surface contaminants, after which they were frozen using liquid nitrogen and stored at -80 °C until the extraction of muscle proteins.

### Meat quality assessment

#### pH measurement

The pH measuring at different time points of slaughter before frozen using liquid nitrogen. pH was measured using a portable pH meter (Testo® 230 m, Testo GmbH & Co., Lenzkirch, Germany) as described by Stadnik & Dolatowski^10^. The electrode was calibrated with standard buffer solutions at 4.0 and 7.0 pH values (Mallinckrodt Chemicals, Phillipsburg, NJ, USA). Points 144 through which the electrode were inserted for measurement were randomly selected.

#### Warner–Bratzler shear force

WBSF measurements of cooked meat (2.54 cm-thick) samples were made based on the methods previously described by Koohmaraie, Shackelford, and Wheeler^11^. Briefly, transverse LL muscle sections were cooked in a water bath until the center was heated to 70 °C, after which they were cooled to under 30 °C. We then extracted core samples (1.27 cm, parallel to the longitudinal fibers) from each LL sample, and peak force was measured with a V-shaped shear blade with a cross-head speed of 400 mm/min.

### Protein isolation

Tissue samples were homogenized using lysis buffer (4% SDS, 1 mM DTT, 150 mM Tris–HCl pH 8.0) followed by a three-minute incubation in boiling water as described previously^12^. Samples were then subjected to sonication on ice, and the resultant crude extract was again incubated in boiling water. Samples were then centrifuged for 10 min at 16,000*g* at 25 °C, and a BCA assay (Beyotime) was used to measure supernatant protein contents.

### iTRAQ analysis

The filter-aided proteome preparation (FASP) approach was used for protein digestion^12^, followed by the iTRAQ labeling of the resultant peptides (Applied Biosystems). Briefly, we combined 30 μL of SDT buffer samples with 200 μg aliquots of individual samples, followed by the use of UA buffer (8 M Urea, 150 mM Tris–HCl pH 8.0) for repeated ultrafiltration. Next, 100 μL iodoacetamide (0.05 M) in UA buffer was added and samples were allowed to rest for 20 min while protected from light, followed by the filters being washed thrice with 100 μL each of UA and DS (50 mM TEAB, pH 8.5) buffers. Next, samples were incubated overnight together with 2 μg of trypsin in 40 μL of DS, followed by another round of filtration. Sample peptide concentrations were then gauged based on absorbance at 280 nm. For the final analysis, random samples were selected from these 20 Yushu and Gannan yak samples and were used for testing. Samples were labeled as follows using iTRAQ reagents in 70 μL of ethanol, after which samples were multiplexed and vacuum-dried: (Yushu-12 h)-113, (Yushu-12 h)-114, (Yushu-72 h)-115, (Yushu-72 h)-116, (Gannan-12 h)-117, and (Gannan-12 h)-118, (Gannan-72 h)-119, (Gannan-72 h)-121.

### Peptide fractionation

Strong cation-exchange (SCX) chromatography (AKTA system, GE Healthcare)^12^ was used to fractionate the labeled peptides prepared for iTRAQ analysis. Briefly, peptides were reconstituted using buffer A (10 mM monobasic potassium phosphate in 25% ACN, pH 2.7) followed by elution with a PolyLC PolySULFOETHYL column at 1 mL/min using buffer B (500 mM potassium chloride, 10 mM monobasic potassium phosphate in 25% ACN, pH 2.7) with the following gradient settings: 0–10% for 2 min, 10–20% for 25 min, 20–45% for 5 min, and 50–100% for 5 min. Resultant fractions were pooled, desalted with Sigma Empore™ C18 Cartridges (I.D. 7 mm, 3 mL, concentrated, and reconstituted using 0.1% (v/v) TFA.

### Phosphopeptide enrichment

Initially, phosphopeptides were subjected to a 40-min agitation step in 500 µL of loading buffer (2% glutamic acid/65% acetonitrile/2% TFA) containing TiO_2_ beads, after which samples were spun for 1 min at 5000 g and beads were recovered^12^. This was then repeated using the supernatant from this initial enrichment analysis, with the two resultant bead samples then being pooled, rinsed using 50 µL volumes of washing buffer I (30% acetonitrile/3% TFA) and II (80% acetonitrile/0.3% TFA), eluted with 50 µL 40% ACN/15% NH4OH, and lyophilized.

### Mass spectrometry

A C18-reversed-phase column (15 cm long, 75 μm I.L., RP-C18 3 μm) was used to elute 10 µL of a combined mixture of 5 µL phosphopeptide solution and 15 µL 0.1% TFA. Buffer B (80% ACN and 0.1% HCOOH) was used for elution at 250 nL/min for 240 min using the following gradient conditions: 0%–60% from 0 to 200 min, 60%–100% from 200 to 216 min, 100% from 216 to 240 min. Survey scans and HCD spectra were obtained at resolutions of 70,000 and 17,500 at m/z 200, respectively. Positive-ion mode was used for mass spectra acquisition using the precursor ions that were most prevalent in initial survey scans. Predictive Automatic Gain Control was used for target value estimation, with a 40.0 s dynamic exclusion duration.

### Data analysis

MASCOT v2.2 (Matrix Science, UK) and Proteome Discoverer v1.4 (Thermo Electron, USA) were used for mass spectra analyses based on comparisons with the Uniport bovine 32,293 20,180,603 database (including 32,293 sequences; downloaded at 20,160,603). For analysis purposes, settings used were as follows: 20 ppm Peptide mass tolerance; 0.1 Da MS/MS tolerance; 2 missed cleavages; Fixed modification of Carbamido methyl (C), iTRAQ8plex (K), and iTRAQ8plex (N-term); Variable modifications of Oxidation (M) and Phosphorylation (S/T/Y); FDR (False discovery rate) ≤ 0.01. Optimal peptide spectrum matches (PSMs) were identified based on pRS scores > 50, with scores > 75% corresponding to a phosphorylation event. Differential protein expression was detected via comparisons between groups, with samples being compared via Student’s *t* tests with a *P* < 0.05 significance threshold. Proteins with multiple changes (*P* > 1.5 or *P* < 0.883) were considered to be differentially enriched in individual samples. Up-regulated and down-regulated proteins were colored in orange and green, respectively, in downstream analyses.

### Bioinformatics analysis

Differentially expressed proteins (DEPs) were annotated using the GO Blast2GO (https://www.blast2go.com/) analysis tool, while KEGG pathway enrichment was evaluated using KAAS (http://www.genome.jp/kaas-bin/kaas_main). Interactions between proteins were evaluated with the STRING database (http://string-db.org/) based on a minimum interaction score cut-off of 0.400. Predicted interactions in this database are based on either direct or indirect evidence from prior studies, co-expression analyses, and genomic/high-throughput analyses.

### Ethical approval

Animals used in the experiment process was to follow the national slaughter and processing Standardization Technical Committee (SAC/TC516).

## Results

### Sample quality changes during postmortem storage

We began by comparing the quality of meat samples isolated from 10 yaks per group over the course of postmortem storage by measuring sample pH values at different time points. We observed significant differences in the pH decline dynamics when comparing samples from Yushu and Gannan yaks (*P* < 0.05) (Table 1). Specifically, at 72 h post-slaughtering, tissue pH values in samples from Yushu yaks were significantly lower than those in samples from Gannan yaks, although in both cases the muscle pH values did decline rapidly over the course of the 72 h postmortem storage period.

**Table 1:** Changes in quality characteristics in Gannan Yak and Yushu Yak LL muscle tissues during postmortem aging at 4 °C (0, 12, 24, 72, 120, and 168 h).

Parameter	Category	Postmortem aging days					
		0 h	12 h	24 h	72 h	120 h	168 h
pH	Gannan	6.55 ± 0.18^aA^	5.32 ± 0.15^Ba^	5.58 ± 0.12^cA^	5.52 ± 0.11^cA^	5.61 ± 0.17^bA^	5.61 ± 0.17^bA^
	Yushu	6.58 ± 0.13^aA^	5.87 ± 0.11^bA^	5.67 ± 0.15^cA^	5.55 ± 0.17^dA^	5.65 ± 0.12^bA^	5.68 ± 0.13^bA^
WBSF/kg	Gannan	4.69 ± 0.56^aA^	5.09 ± 0.27^aA^	6.53 ± 0.18^cA^	5.43 ± 0.15^bA^	5.19 ± 0.29^bA^	4.79 ± 0.12^aA^
	Yushu	5.20 ± 0.46^aB^	5.29 ± 0.15^aB^	6.88 ± 0.09^cB^	5.79 ± 0.15^bB^	5.53 ± 0.23^bB^	4.96 ± 0.32^aB^

^a-e^Means without shared superscripts in a row differed significantly (*P* < 0.05).

^A-B^Means without shared superscripts in a column for a given parameter differed significantly (*P* < 0.05).

We additionally examined changes in WBSF values over the course of storage in these two sets of yak samples (Table 1). This analysis revealed significant differences in tenderness changes between Yushu and Gannan yak samples after 12 h of storage (*P* < 0.05), but values remained unchanged after 72 h. After 72 h, further reductions in WBSF values were observed. Prior studies have found that other low pH muscle fillets exhibited better tenderness but lower water retention capacity^13,14^.

### Bioinformatics analysis

#### Proteomic profile changes over the course of storage

We next leveraged the iTRAQ technology and LC–MS/MS in order to quantify proteomic changes within yak LL muscle samples during storage. In total we identified 1899 DEPs when making the following comparisons: Yushu 12 h versus Gannan 12 h, Yushu 72 h versus Gannan 72 h with *P* < 0.05 and a quantitative ratio of > 1.2 or < 0.833 as cut-off criteria (Table 2). When comparing the two yak samples collected at 12 h, we identified 290 DEPs (245 down-and 45 up-regulated), while a comparison of the two samples revealed 436 DEPs (56 down- and 380 up-regulated).

**Table 2:** Numbers of Differentially Expressed Proteins (quantitative ratio > 1.2 or < 0.833, *P* < 0.05).

	Upregulation of Protein Quantity	Down-regulation of protein quantity	Total number of differentially expressed proteins
Yushu 12 h versus Gannan 12 h	45	245	290
Yushu 72 h versus Gannan 72 h	380	56	436

#### Functional enrichment analysis

**Figure 1:**
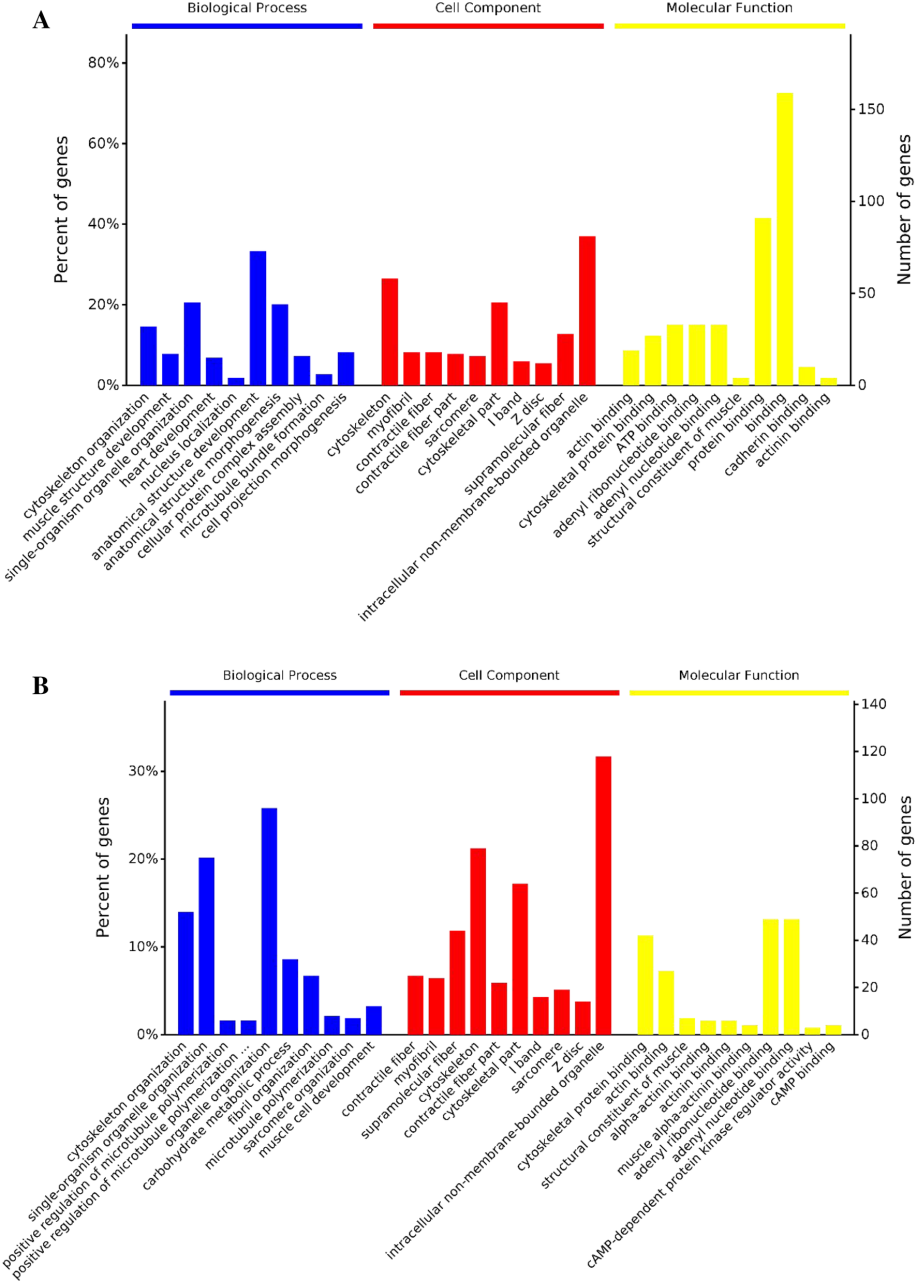
(**A**) A comparison of functional classification of identified phosphoproteins at 12 h. (**B**) A comparison of functional classification of identified phosphoproteins at 72 h.

**Figure 2:**
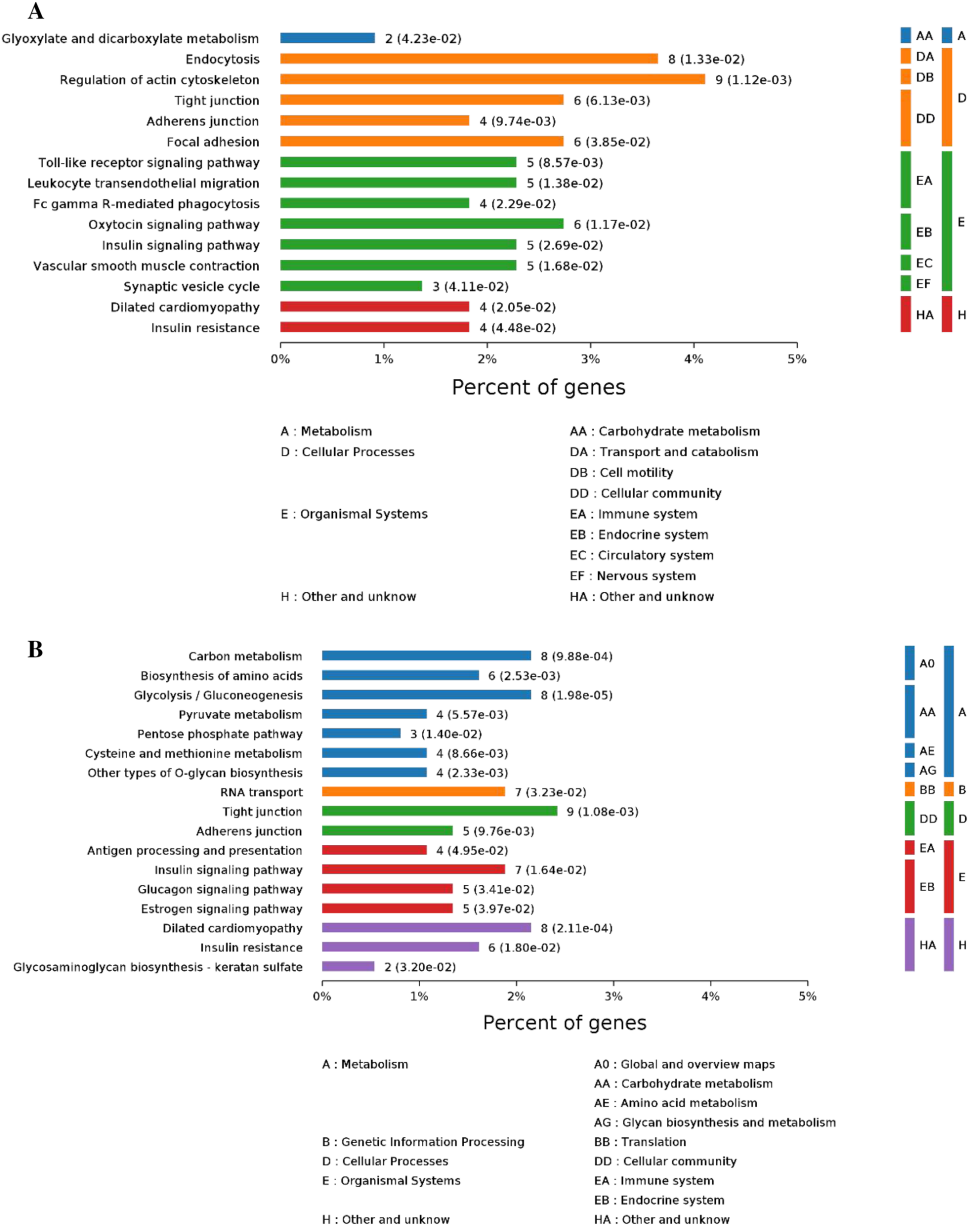
(**A**) Enriched KEGG pathways associated with the comparison of Yushu 12 h versus Gannan 12 h samples. (**B**) Enriched KEGG pathways associated with the comparison of Yushu 72 h versus Gannan 72 h samples.

**Figure 3:**
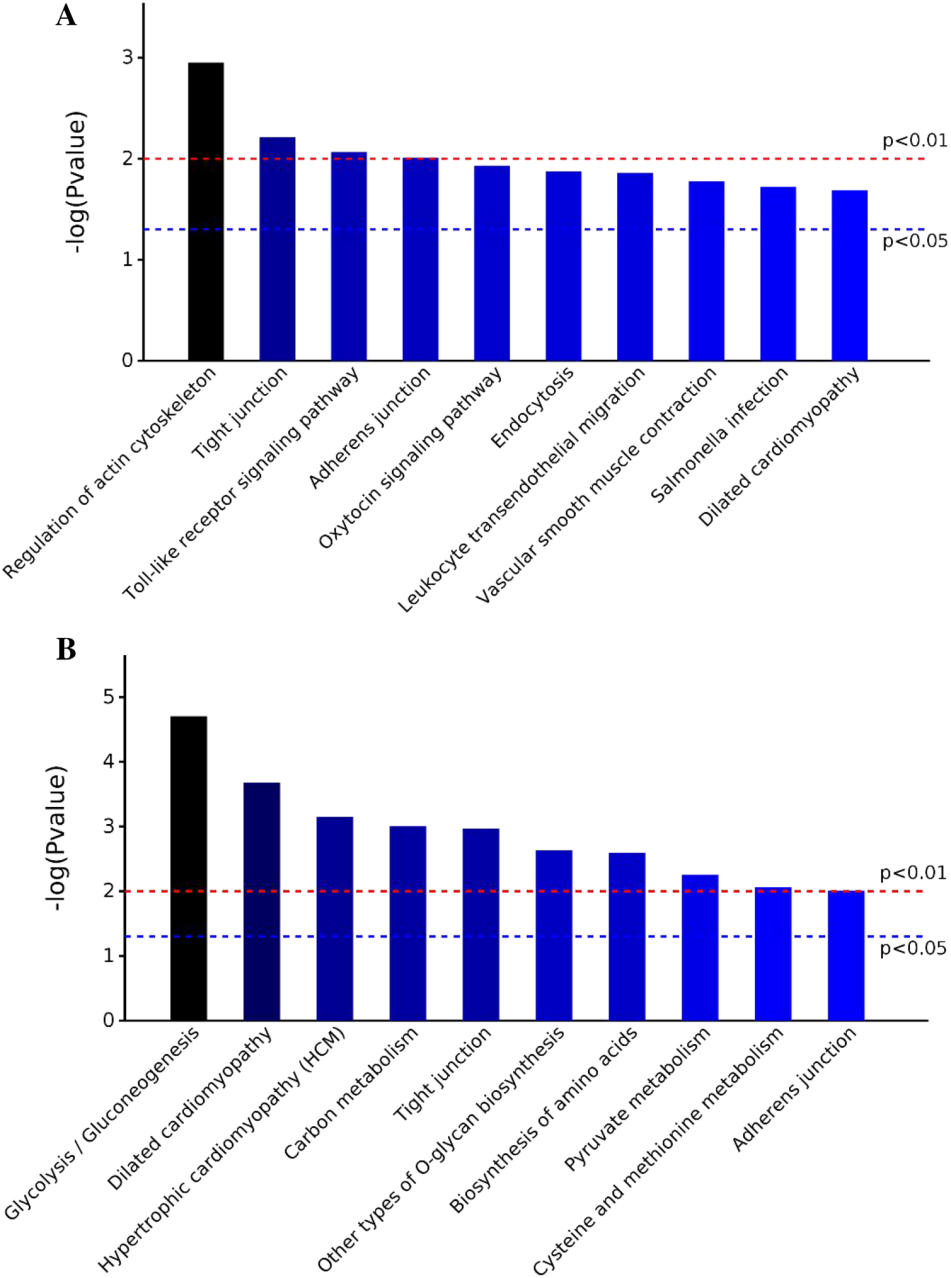
(**A**): Distributions of enriched KEGG pathways associated with the comparison of Yushu 12 h versus Gannan 12 h samples. (**B**) Distributions of enriched KEGG pathways associated with the comparison of Yushu 72 h versus Gannan 72 h samples.

In order to explore the biological functionality of the differentially expressed phosphoproteins identified in the above analysis, we next conducted GO and KEGG functional enrichment analyses of these DEPs (Figs. 1, 2, 3). When comparing Yushu and Gannan yak samples collected after 12 h, we identified 2837 enriched biological processes (316 significant), 463 enriched cellular components (132 significant), and 579 enriched molecular functions (107 significant). For full details regarding these GO terms and those associated with the 72 h samples, see the Supplementary materials.

DEPs in these analyses were associated with GO terms including cytoskeleton organization (GO:0007010), muscle structure development (GO:0061061), heart development (GO:0007507), actin cytoskeleton organization (GO:0030036), positive regulation of cytoskeleton organization (GO:0051495), glucan metabolic process (GO:0044042), glycogen metabolic process (GO:0005977), cellular glucan metabolic process (GO:0006073), energy reserve metabolic process (GO:0006112), tripeptide transmembrane transport (GO:0035443), and cellular polysaccharide metabolic process (GO:0044264).

In a KEGG enrichment analysis, we found these DEPs to be primarily enriched in pathways including the regulation of the actin cytoskeleton (bta04810), leukocyte transendothelial migration (bta04670), the dilated cardiomyopathy insulin signaling pathway (bta05414), starch and sucrose metabolism (bta00500), the cAMP signaling pathway (bta04024), adrenergic signaling in cardiomyocytes (bta04261), butanoate metabolism (bta00650), propanoate metabolism (bta00640), galactose metabolism (bta00052), the citrate cycle (TCA cycle) (bta00020), the glucagon signaling pathway (bta04922), and pyruvate metabolism (bta00620). These annotation results suggest that these DEPs are particularly associated with glycolysis-related pathways including pyruvate metabolism, the pentose phosphate pathway, and energy metabolism.

### Protein–protein interactions

**Figure 4:**
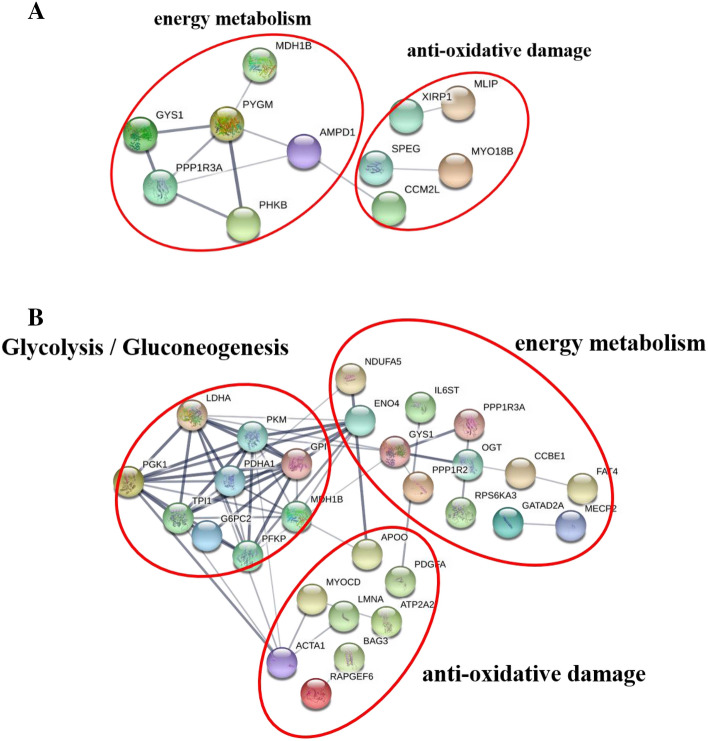
(**A**) A Protein–Protein Interaction Network Corresponding to the Comparison of Yushu and Gannan yak samples at 12 h. This network view summarizes the predicted associations for a particular group of proteins. The network nodes are proteins, while the edges represent predicted functional associations. Edges may be drawn with any of 7 differently colored lines corresponding to the seven types of evidence used in predicting these associations. A red line indicates the existence of fusion evidence; a green line—neighborhood evidence; a blue line – co-occurrence evidence; a purple line—experimental evidence; a yellow line—text mining evidence; a light blue line—database evidence; a black line—coexpression evidence. (**B**): A Protein–Protein Interaction Network Corresponding to the Comparison of Yushu and Gannan yak samples at 72 h.

A protein–protein interaction network of differentially abundant proteins identified through the above analyses is shown in Fig. 4A,B. Proteins are represented as network nodes, while the edges represent the predicted functional associations between these proteins. The interactions between the imported proteins and all proteins stored in the database were then identified. In cells, proteins construct complex networks to execute their functions through protein–protein interactions, modifications, and other regulatory relationships. In agreement with our iTRAQ proteomic analysis, glycolysis, energy metabolism, and hypoxia adaptability were all associated with core proteins in this network. This confirmed that glycolysis, energy metabolism, and hypoxia adaptability were closely related to cooking loss. The associated proteins are likely to play key roles in the correlations between postmortem aging and yak meat tenderness.

### Quantitative phosphopeptide analysis

Based on our iTRAQ analysis, we were additionally able to identify differences in phosphorylation levels of particular DEPs identified when comparing Yushu and Gannan yak samples at 12 and 72 h post-slaughtering. We were able to identify more than one phosphopeptide and phosphosite in 7 glycolytic rate-, 39 energy metabolism- and 12 hypoxic adaptation-related phosphoproteins (Fig. 4A,B), highlighting clear differences in phosphorylation status between these samples.

## Discussion

### Protein phosphorylation events are associated with changes in muscle tenderness

Three main factors influence the tenderness of beef. First, the type and proportion of muscle fibers in a given muscle can profoundly alter such tenderness, with tenderness being negatively correlated with the proportion of rapidly oxidized glycolytic fibers in a given muscle^15^. Second, the glucose metabolism pathway is related to the intramuscular synthesis of fatty acids, which directly impacts muscle tenderness^16^. Third, anaerobic glycolysis and associated lactic acid production can profoundly alter changes in muscle tone and volume after slaughter, thereby influencing the tenderization rate therein^17,18^. After animals are slaughtered, intramuscular blood and oxygen supplies are interrupted and muscles undergo switch from aerobic metabolism to anaerobic glycolysis. As such, the tenderization of the muscles after slaughter is largely dependent on the glycolysis pathway and on a series of physiological and biochemical changes. This increased reliance upon anaerobic glycolysis is accompanied by the accumulation of lactic acid products, resulting in a decrease in intramuscular pH. This pathway facilitates myofibril degradation and further improves tenderness after slaughter^17–19^.

We found that beef isolated from Yushu yaks exhibited a relatively strong glycolytic ability after slaughter, in turn resulting in a rapid decline in pH that can lead to the degeneration of muscle proteins and excessive muscle contraction, increasing overall toughness. In contrast, in Gannan yaks the intramuscular glycogen reserves were insufficient, and as such, anaerobic fermentation was limited after slaughter, resulting in the more rapid termination of glycolysis and more rapid onset of rigor mortis but a lower degree of overall muscle contraction relative to Yushu yaks. Over time, glycolytic enzyme activity decreases as these enzymes are hydrolyzed and their expression levels fall. In higher pH environments many proteolytic enzymes will become activated, including the highly hydrolytic μ-calpain enzyme that can be rapidly engaged in Gannan yak muscle following slaughter, leading to more efficient protein hydrolysis in this setting. Fibrillin and many key glycolytic enzymes are also hydrolyzed over time by these proteolytic enzymes, with such hydrolysis being directly related to muscle tenderization^15,20,21^. As cathepsin activity was relatively strong in Yushu yak muscles, this likely leads to more complete myofibril degradation and improved tenderness. Laville et al. also surveyed the beef proteome in order to identify correlates for tenderness, and they found that glycolytic enzymes were quickly engaged following slaughter and were directly involved in the tenderization process. Glycolytic enzymes such as PGK1 and GAPDH degrade faster in tender meat, leading to the presence of a large number of related degradation fragments^22^. Lametsch et al. also assessed post-mortem pork samples and found that the degradation of CK, PKM, GP was correlated with the degradation of myosin, actin, and troponin-T such that they were reliable biomarkers of muscle tenderness^23,24^.

### Phosphorylation of glycolysis related proteins

Among the major differentially detected proteins associated with the glycolytic pathway, we identified phosphoglucomutase-1 isoform X1 (*PGM1*), Triosephosphate isomerase (*TIM/TPI1*), phosphoglycerate kinase (*PGK1*), phosphoglycerate mutase2 (*PGAM2*), pyruvate kinase isozymes M1/M2 isoform X1 (*PKM1/M2*), L-lactate dehydrogenase A chain (*LDHA*), creatine kinase M chain (*CKM*), glycerol-3-phosphate dehydrogenase (*GPDH*), hydroxyacylglutathione hydrolase (*HAGH*), glycol phosphorylase (*GP*), lactoylglutathione lyase (*LGUL*). The expression of these key glycolytic enzymes was related to post-mortem pH changes, indicating that post-mortem glycolytic enzyme expression is associated with both the rate and the degree of intramuscular pH decline. In contrast, we found that enzymes involved in the TCA cycle were such as the dihydrolipoyllysine-residue acetyltransferase component of pyruvate dehydrogenase complex, mitochondrial isoform X1 (*DLAT*), malate dehydrogenase, mitochondrial isoform X1 (*MDH2*), pyruvate dehydrogenase E1 component subunit beta, and mitochondrial precursor (*PDHB*) were expressed at higher levels in the muscles of yaks raised at higher altitudes. These proteins are primarily involved in the process of mitochondrial respiration, and their elevated levels suggest a greater mitochondrial respiration capacity in the tissues of these animals after slaughter. We observed reduced levels of phosphoglucomutase-1 isoform X1 (*PGM1*), which is involved in the pentose phosphate pathway, in the muscles of yaks raised at higher altitudes^25,26^. We also found that oxidative phosphorylation pathway-related proteins including succinate dehydrogenase [ubiquinone] iron-sulfur subunit, mitochondrial isoform X1 (*SDHB*), cytochrome c oxidase subunit 5A, mitochondrial isoform X1 (*COX5A*), cytochrome c oxidase subunit 5B, mitochondrial precursor (*COX5B*), synthase subunit alpha, mitochondrial isoform X1 (*ATP5A*), ATP synthase subunit beta, mitochondrial precursor (*ATP5B*), ATP synthase subunit d, and mitochondrial isoform X2 (*ATP5D*) were all abundantly expressed in high-altitude yak samples^27^. In addition, we identified differentially abundant proteins associated with the fructose/mannose metabolic pathway and with the pyruvate metabolism pathway. Overall, our results suggest that these differentially abundant proteins can participate in a number of post-mortem muscle changes through their respective metabolic pathways, thereby impacting time-dependent changes in meat quality.

### Energy metabolism pathways

We identified 290 and 436 DEPs when comparing Gannan and Yushu yak samples at 12 and 72 h postmortem, respectively. We detected both structural and stress proteins among these DEPs, but we found that energy metabolism-related proteins were the most common proteins detected in this analysis. This allowed us to conclude that Gannan yaks had higher energy metabolism rates than did Yushu yaks at 72 h post-slaughter.

During fructose metabolic processing, both fructose bisphosphatase C-A and triose bisphosphatase are required in sequence, and we found that both were expressed at higher levels in Gannan yaks. The metabolism of fructose-6-phosphate occurs early during glycolysis. Upon glycolytic processing, glucose, which is relatively stable, is first activated via ATP-mediated phosphorylation to yield a less stable glucose-6-phosphate molecule^25^. Glucose-6-phosphate is in turn rearranged by the glucose phosphate isomerase enzyme to yield fructose-6-phosphate. In total, two ATP molecules are consumed per glucose molecule so as to yield fructose-1 and 6-diphosphate. These molecules in turn are metabolized to produce glyceraldehyde-3-phosphate and dihydroxyacetone-3-phosphate, with the latter then being converted into an additional glyceraldehyde 3-phosphate. Glyceraldehyde-3-phosphate is then oxidized to produce 1,3-diphosphoglyceride, which can release two electrons and one H^+^, and can be transferred to the electron acceptor NAD^+^, generating NADH and transferring the released energy into a high-energy phosphate bond. The loss of the high-energy phosphate bond in unstable 1,3-diphosphoglyceride yields 3-phosphoglyceric acid, with the released energy being stored as an ATP molecule. Next, 3-PGA is rearranged into 2-PGA, which undergoes dehydration to yield phosphoenolpyruvate (PEP). Lastly, PEP transfers a phosphate group to ADP to yield ATP and pyruvate^28^.

In addition, certain mitochondrial-related enzymes including *DLAT, MDH2, PDHB, SDHB, COX5A, COX5B, ATP5A, ATP5B,* and *ATP5D* are involved in the process of oxidative respiration, and were expressed at higher levels in Yushu yak samples. This suggests that beef prepared from high-altitude yaks exhibits more robust oxidative metabolism after slaughter. In living animals, ATP is continuously produced and supplied primarily via mitochondrial oxidative phosphorylation, with the energy consumed by this process being mainly dependent upon the oxidative decomposition of fatty acids, glucose, and glycogen^29^. When tissue energy metabolism is vigorous, large quantities of oxygen must be consumed in order to support this oxidative metabolism. When tissues are hypoxic, ATP production is instead supplemented by glycolytic processes within the cytoplasm of cells^30^. The yaks in the present study may have different glycogen reserves as a result of their exposure to different stresses prior to slaughter, resulting in differing post-slaughter patterns of energy metabolism in these animals. High-altitude yak muscle samples exhibited more robust strong oxidative metabolism, reduced intramuscular and surface oxygen concentrations, and differential oxygenation. Myoglobin deoxygenation can result in darker meat coloration^28^.

### Regulation of protein kinase activity

During the postmortem aging and storage process, calpastatin can become degraded, with the rate of such degradation being closely associated with meat tenderization and proteolysis rates^31^. We observed reduced calpastatin levels in meat samples as storage time was increased, suggesting that calpains may play a greater role in muscle structural protein breakdown at later time points during storage. Indeed, several studies have highlighted roles for calpains in the regulation of skeletal muscle tenderness^32–34^.

Other enzymes also control the tenderization process, such as 5′-nucleotidase which can hydrolyze extracellular nucleotides such that they become membrane permeable nucleosides, yielding a phosphate in the process. Several studies have suggested that muscle phosphorylation is positively correlated with meat tenderness^33^, and as such 5’-nucleotidase may play a role in the regulation of early-stage changes in meat tenderness. Another enzyme, ADP/ATP translocase 1, catalyzes the exchange of cytoplasmic ADP with mitochondrial ATP across the mitochondrial inner membrane, utilizing phosphate molecules in the process and thus functioning in a manner opposed to the activity of 5annucleotidase. This raises the possibility that ADP/ATP translocase 1 may function primarily during later stages of the meat tenderization process. Myosin light chain kinase regulates yak muscle contraction via phosphorylating myosin light chain molecules, with such phosphorylation positively impacting meat tenderness^33^. As such, all three of these enzymes are involved in phosphorylation-related activities in yak muscle tissue. Studies demonstrated that the phosphorylation of *MyLC2* occurs during beef rigor mortis, while D'Alessandro et al. similarly demonstrated that phosphorylation plays a key role in the progression of Chianina Bos taurus *longissimus dorsi* from muscle into meat^35^. Our findings further indicate that these phosphorylation-related proteins are closely linked to smooth muscle tenderness, and we thus hypothesize that yak muscle protein phosphorylation may play a vital role in muscle tenderization.

## Conclusions

In summary, our results offer novel insights into the proteomic changes that occur during the postmortem aging of the yak LL muscle. We identified 290 DEPs at 12 h and 436 DEPs at 72 h when comparing Gannan and Yushu yak samples. Bioinformatics analyses suggested that these DEPs are associated with cell structure, the glycolytic pathway, and energy metabolism. Further research regarding the post-translational modification of these proteins and associated changes in metabolite levels are still required.

## Supplementary Information

Supplementary Information.

## Data Availability

All data, models, and code generated or used during the study appear in the submitted article.

## References

[1] Goda N, Kanai M (2012). Hypoxia-inducible factors and their roles in energy metabolism. Int. J. Hematol..

[2] Muralimanoharan S, Maloyan A, Myatt L (2015). Mitochondrial function and glucose metabolism in the placenta with gestational diabetes mellitus. Placenta.

[3] Zuo HX, Han L, Yu Q (2017). Proteomics and bioinformatics analyses of differentially expressed proteins in yak and beef cattle muscle. Trans. Chin. Soc. Agric. Mach..

[4] Hardie DG, Scott JW, Pan DA, Hudson ER (2003). Management of cellular energy by the AMP-activated protein kinase system. FEBS Lett..

[5] Hardie DG, Ross FA, Hawley SA (2012). AMPK: A nutrient and energy sensor that maintains energy homeostasis. Nat. Rev. Mol. Cell Biol..

[6] Li Z, Li M, Du M, Shen QW, Zhang D (2018). Dephosphorylation enhances postmortem degradation of myofibrillar proteins. Food Chem..

[7] Yang Y, Han L, Yu Q (2020). Phosphoproteomic analysis of longissimus lumborum of different altitude yaks. Meat Sci..

[8] Yayuan Y, Rende S, Ling H, Gao Yongfang Yu, Qunli MJ (2018). Changes of AMPK activity and energy metabolism of postmortem beef at different altitudes. Trans. Chin. Soc. Agric. Mach..

[9] René L, Emøke B (2001). Proteome analysis applied to meat science: Characterizing post mortem changes in porcine muscle. J. Agric. Food Chem..

[10] Stadnik J, Dolatowski ZJ (2011). Influence of sonication on Warner–Bratzler shear force, colour and myoglobin of beef (m.semimembranosus). Eur. Food Res. Technol..

[11] Koohmaraie, M., Shackelford, S. D., & Wheeler, T. L. Effects of a beta-adrenergic agonist (l-644,969) and male sex condition on muscle growth and meat quality of callipyge lambs. *J Anim Sci.* **74**(1), 70–79 10.2527/1996.74170x (1996). 10.2527/1996.74170x 8778114

[12] Wisniewski JR, Zougman A, Nagaraj N (2009). (2009) Universal sample preparation method for proteome analysis. Nat. Methods.

[13] Watanabe, A., Daly, C. C., Devine, C. E. The effects of the ultimate pH of meat on tenderness changes during ageing. *Meat Sci.* **42**(1), 67–78 10.1016/0309-1740(95)00012-7 (1996). 10.1016/0309-1740(95)00012-7 22060302

[14] Bowker B, Zhuang H (2015). Relationship between water-holding capacity and protein denaturation in broiler breast meat. Poult. Sci..

[15] Dransfield E (1994). Modelling post-mortem tenderization-V: Inactivation of calpains. Meat Sci..

[16] Wood J, Enser M, Fisher A, Nute G, Sheard P, Richardson R, Hughes S, Whittington F (2008). Fat deposition, fatty acid composition and meat quality: A review. Meat Sci..

[17] Sentandreu MA, Coulis G, Ouali A (2002). Role of muscle endopeptidases and their inhibitors in meat tenderness. Trends Food Sci. Technol..

[18] Ouali A, Herrera-Mendez CH, Coulis G, Samira B, Boudjellal A, Harhoura K, Aubry L, Sentandreu MA (2007). Meat tenderisation and muscle cell death, two highly related events. Tehnolog. Mesa.

[19] Sawdy CJ, Kaiser AS, St PRN, Wick PM (2004). Myofibrillar 1-D fingerprints and myosin heavy chain MS analyses of beef loin at 36 h postmortem correlate with tenderness at 7 days. Meat Sci..

[20] Uytterhaegen L, Claeys E, Demeyer D (1992). The effect of electric stimulation on beef tenderness, protease activity and myofibrillar protein fragmentation. Biochemistry.

[21] Uytterhaegen L, Claeys E, Demeyer D (1994). Effects of exogenous protease effectors on beef tenderness development and myofibrillar degradation and solubility. J. Anim. Sci..

[22] Laville E, Sayd T, Morzel M, Blinet S, Chambon C, Lepetit J (2009). Proteome changes during meat-aging in tough and tender beef suggest the importance of apoptosis and protein solubility for beef aging and tenderization. J. Agric. Food Chem..

[23] Lametsch R, Roepstorff P, Bendixen E (2002). Identification of protein degradation during post-mortem storage of pig meat. J. Agric. Food Chem..

[24] Lametsch R, Karlsson A, Rosenvold K, Andersen HJ, Roepstorff P, Bendixen E (2003). Postmortem proteome changes of porcine muscle related to tenderness. J. Agric. Food Chem..

[25] Anderson MJ, Lonergan SM, Huff-Lonergan E (2012). Myosin light chain 1 release frommyofibrillar fraction during postmortemaging is a potential indicator of proteolysis and improvement in tenderness of beef. Meat Sci..

[26] Bouley J, Chambon C, Picard B (2004). Mapping of bovine skeletal muscle proteins using two-dimensional gel electrophoresis and mass spectrometry. Proteomics.

[27] Barbut S, Sosnicki AA, Lonergan SM, Knapp T, Ciobanu DC, Gatcliffe LJ, Huff-Lonergan E, Wilson EW (2008). Progress in reducing the pale, soft, and exudative (PSE) problemin pork and poultry meat. Meat Sci..

[28] Maeda K, Yamamoto F, Toyoshi M, Irie M (2014). Effects of dietary lysine/protein ratio and fat levels on growth performance and meat quality of finishing pigs. Anim. Sci. J..

[29] Calabrò S, Cutrignelli MI, Gonzalez OJ, Chiofalo B, Grossi M, Tudisco R (2014). Meat quality of buffalo young bulls fed faba bean as protein source. Meat Sci..

[30] L. Chen, Li, Z., & Everaert, N. *et al.* Quantitative phosphoproteomic analysis of ovine muscle with different postmortem glycolytic rates. *Food Chem*. **280**, 203–209. (2019) 10.1016/j.foodchem.2018.12.056 30642488

[31] Laville E, Sayd T, Morzel M, Blinet S, Chambon C, Lepetit J (2009). Proteome changes during meat aging in tough and tender beef suggest the importance of apoptosis and protein solubility for beef aging and tenderization. J. Agric. Food Chem..

[32] Geesink GH (2006). calpain is essential for postmortem proteolysis of muscle proteins. J. Anim. Sci..

[33] Koohmaraie M, Doumit ME, Wheeler TL (1996). Meat toughening does not occur when rigor shortening is prevented. J. Anim. Sci..

[34] Lana A, Zolla L (2016). Proteolysis in meat tenderization from the point of view of each single protein: A proteomic perspective. J. Proteom..

[35] D'Alessandro A, Zolla L (2013). Meat science: From proteomics to integrated omics towards system biology. J. Proteom..

